# Microfluidic Rheometry and Particle Settling: Characterizing the Effect of Polymer Solution Elasticity

**DOI:** 10.3390/polym14040657

**Published:** 2022-02-09

**Authors:** Salah A. Faroughi, Francesco Del Giudice

**Affiliations:** 1 Geo-Intelligence Laboratory, Ingram School of Engineering, Texas State University, San Marcos, TX 78666, USA; 2 Department of Chemical Engineering, Faculty of Science and Engineering, School of Engineering and Applied Sciences, Swansea University Bay Campus, Fabian Way, Swansea SA1 8EN, UK; francesco.delgiudice@swansea.ac.uk

**Keywords:** viscoelasticity, particle settling, dilute polymeric solutions, Oldroyd-B model, microfluidic rheometry, drag coefficient, hydraulic fracturing

## Abstract

The efficient transport of solid particles using polymeric fluids is an important step in many industrial operations. Different viscoelastic fluids have been designed for this purpose, however, the effects of elasticity have not been fully integrated in examining the particle-carrying capacity of the fluids. In this work, two elastic fluid formulations were employed to experimentally clarify the effect of elasticity on the particle drag coefficient as a proxy model for measuring carrying capacity. Fluids were designed to have a constant shear viscosity within a specific range of shear rates, $\dot{\gamma }<50\;\left(1/s\right)$, while possessing distinct (longest) relaxation times to investigate the influence of elasticity. It is shown that for dilute polymeric solutions, microfluidic rheometry must be practiced to obtain a reliable relaxation time (as one of the measures of viscoelasticity), which is on the order of milliseconds. A calibrated experimental setup, furnished with two advanced particle velocity measurement techniques and spheres with different characteristics, was used to quantify the effect of elasticity on the drag coefficient. These experiments led to a unique dataset in moderate levels of Weissenberg numbers, 0<Wi<8.5. The data showed that there is a subtle reduction in the drag coefficient at low levels of elasticity (Wi<1), and a considerable enhancement at high levels of elasticity (Wi>1). The experimental results were then compared with direct numerical simulation predictions yielding $R^{2}=0.982$. These evaluations endorse the numerically quantified behaviors for the drag coefficient to be used to compare the particle-carrying capacity of different polymeric fluids under different flow conditions.

## 1. Introduction

**Figure 1:**
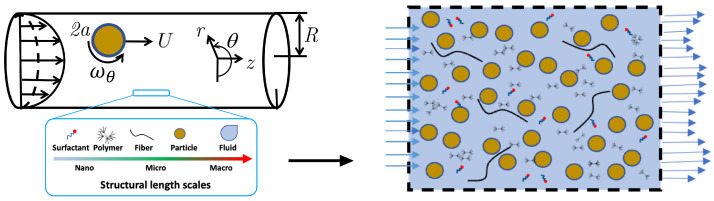
Schematic of sand particles transport in hydraulic fracturing operation where hundreds of millions of sand particles are co-injected alongside fracturing fluids (e.g., dilute polymeric and surfactant solutions with/out fibers) to preserve the conductivity of the induced fracture networks after the pressure release.

The dynamics of solid particles flowing through polymeric fluids is strongly affected by viscoelasticity of the fluid [1,2]. Elasticity of the fluid, in addition to viscosity, is an integral element to consider when designing efficient solid particle transport in many advanced manufacturing and industrial operations, such as processing of highly-filled viscoelastic polymer melts and elastomers [3,4], processing of semi-solid conductive flow battery slurries [5], cement slurries flow [6], and biological applications like the flow-induced migration of circulating cancer cells in biopolymeric media such as blood [7]. Hydraulic fracturing operations in tight oil and gas fields [8] is another important application of particle-laden polymeric fluids. As shown in Figure 1, in hydraulic fracturing hundreds of millions of sand particles (also known as proppant) are co-injected alongside fracturing fluids (e.g., dilute polymeric and surfactant solutions with/out fibers) to preserve the conductivity of the induced fracture networks after the pressure release [9,10].

Due to the lack of physics and theoretical models, or computational power, state-of-the-art fracturing simulators ignore the relevance of the flow properties (i.e., elastic response under an external flow) of polymeric fluids [2,11,12,13]. Some attempts have been made in this direction for particle-free polymer solutions, however, not in the context of hydraulic fracturing, and for very basic geometrical shapes of the channel [14,15]. Fluid elasticity specifically alters the sedimentation and rotation rate of a particle, which in turn causes different cross-stream flow-induced migration behaviors, affecting the overall particle transport efficiency [15,16,17,18]. There is still a need to fully understand how to tune the properties of polymeric fluids to efficiently transport particles. To fill this gap, not only are the effect of particle shapes and types considered [19,20], but also different carrying polymeric fluids are being formulated [8,21]. For these fluids, considering the importance of polymer type, structure, solubility, and charge, the most common variables to design new solutions are the average polymer molecular weight, $M_{w}$, and the polymer concentration, *c* [22,23]. Dilute polymeric solutions, in this work, refer to a solution with 0.01≤cc*≤1, where $c^{*}$ is the overlap concentration [22,24].

Despite the complexity of such systems, the particle-carrying capacity of a fluid is estimated by mapping the translation of a single sphere in inertia-less steady-state conditions [25,26]. In hydraulic fracturing, the importance of this measuring criterion has been originated by the low shear rate conditions experienced by particles within the fractures after the pressure release. This test has been bench-marked since Sir George Stokes calculated, for the first time, the drag force on a single sphere translating through an unbounded Newtonian fluid. The model introduced by Stokes, however, only accounted for the shear viscosity of the fluid, and required other correction factors to be suitable under different flow conditions or fluid types. In a series of works [17,27,28], Gomma et al. showed that the effective shear viscosity is not the only factor to design efficient particle transport, and the fluid elasticity, quantified via the shear modulus, also plays a significant role. Several researchers [1,23,29,30,31,32] conducted comprehensive experimental and numerical investigations to determine the effect of fluid elasticity on the terminal velocity of a single sphere settling in a non-Newtonian elastic fluid in order to quantify the drag coefficient. These studies, even though they, in some cases, provided contradictory conclusions [11,33], generally showed that the fluid elasticity hinders the particles’ motion, and the effect is more pronounced at a high level of elasticity [2,23].

In many practical cases, because of the strong interactions between elasticity and viscosity of the polymeric fluids, the hindrance due to elasticity was intermingled with the inherent shear-thinning properties, i.e., the fact that the viscosity of the polymeric fluid surrounding the falling sphere decreases during the sphere motion [11]. Blyton et al. [11] formulated different fluids to study the effect of fluid elasticity and shear-thinning individually, at a very high levels of flow elasticity. They concluded that the terminal velocity of a spherical particle in fluids possessing similar shear viscosity profiles decreases significantly with increasing the elasticity. Faroughi et al. [2] conducted direct numerical simulations to construct a correction model for the drag force on a particle translating in dilute polymer solutions with low to moderate levels of elasticity in negligible inertia conditions. This approximate model accounted for the effect of elasticity considering the viscoelastic fluids with constant viscosity, e.g., Boger fluids [34]. This model predicts that the drag coefficient of a particle slightly decreases at low levels of elasticity, but substantially increases at high level of elasticity. The latter is due to the large elastic stresses developing on the surface as well as in the wake of the particle [2]. This approximate drag model thus explained some of the contradictory conclusions generated in previous studies. However, this numerically-driven model itself has not been verified experimentally yet due to the lack of data at moderate level of elasticity, i.e., dilute polymeric solutions.

The current study is undertaken to fulfill two main goals: (i) outline an appropriate scheme to infer the particle-carrying capacity of polymeric fluids, and (ii) generate unique static settling, or drag coefficient, data in moderate levels of elasticity. To this end, first, the theoretical background is briefly reviewed for the particle dynamics and rheology measurements (e.g., conventional and microfluidic rheometry) to accurately determine the parameters required to infer the drag coefficient. Then, two fluid formulations are designed with distinct longest relaxation times to carry out the inertia-less particle settling tests at low to moderate level of elasticity. Next, the experimental setup and velocity measurement procedures are elaborated, and the experimental results are presented and weighted against the approximate drag model developed by Faroughi et al. [2]. Finally, the main conclusions of the work are summarized.

## 2. Theoretical Background

### 2.1. Dimensionless Parameters

The interplay among different mechanisms controlling particle transport can be studied by decoupling different relevant forces acting on particles. The most important ones induced by the viscoelastic fluids are the drag, inertial, and transversal forces for which the theoretical developments are very limited [2]. Dimensionless numbers can be employed here to examine the particle transport behavior highlighting the importance of relevant forces. The viscoelasticity of polymeric fluids can be quantified using Weissenberg number, Wi, defined as,

(1) $$\begin{array}{c} Wi\equiv \lambda \dot{\gamma }=\frac{\lambda U}{a}, \end{array}$$

for a spherical particle with radius *a* settling through the fluid. Here, λ is the longest relaxation time, and $\dot{\gamma }$ represents a characteristic shear rate defined based on the terminal settling velocity, *U*, of the particle. For a Newtonian fluid, the Weissenberg number is Wi=0 corresponding to zero elasticity. A higher Weissenberg number, Wi>0, represents a more pronounced elasticity in the fluid.

The presence of coiled or stretched polymers also impacts the effective shear viscosity of the polymeric fluid through hydrodynamic and physical interactions similar to the presence of a cloud of solid particles [35,36]. This effect can be parameterized using,

(2) $$\begin{array}{c} \zeta =\frac{\eta_{P}}{\eta_{S}+\eta_{P}}=\frac{\eta_{P}}{\eta_{0}}, \end{array}$$

where ζ represents the retardation ratio, $\eta_{P}$ is the polymer contribution to the shear viscosity, $\eta_{S}$ is the solvent contribution to the shear viscosity, and $\eta_{0}=\eta_{P}+\eta_{S}$ is the total shear viscosity in the limit of vanishing shear rate. For constant-viscosity viscoelastic fluids, e.g., Boger fluids [34], the relaxation time and retardation ratio, λ and ζ repetitively, are the two important characteristics that define viscoelastic behaviors. These fluids are generally modeled using the Oldroyd-B constitutive equation [37] that best represents the polymer contribution to the momentum exchange in very dilute polymer solutions at low Weissenberg number. However, many realistic suspending fluids show mid to strong shear-thinning features, leading to more complex and nonlinear dependencies at nonvanishing Weissenberg numbers at which shear-thinning effects become even more pronounced [38]. Several viscoelastic constitutive models have been developed over the past few decades to model such fluids [39,40,41]. Among all, the Giesekus model [42] is generally used to best represent the polymer contribution to the momentum exchange in dilute to semidilute polymer solutions. The Giesekus model is developed based on configuration-dependent molecular mobility. Therefore, the viscoelastic component of the polymeric stress tensor is represented by λ and ζ as well as the mobility factor, α, which theoretically varies between zero and unity (practically between zero and 0.5 [12]) and accounts for the shear-thinning behavior of the polymeric fluids.

Another important dimensionless number is the Reynolds number representing the ratio between inertial and viscous forces, which is defined as,

(3) $$\begin{array}{c} Re=\frac{\rho_{f}Ua}{\eta_{0}}, \end{array}$$

where $\rho_{f}$ is the density of the fluid. Particles experience different flow regimes, i.e., turbulent to creeping flow regimes categorized by Re number, in different operations. The particle static settling experiment, as a method to differentiate the carrying capacity of fluids, has generally been studied at low Reynolds numbers, Re≪1, corresponding to the creeping flow regime [17,23].

### 2.2. Drag Coefficient for Viscoelastic Fluids

For a single particle settling in a viscoelastic fluid, one may carry out the drag coefficient on the surface of the particle using a surface integration of the total stress comprising the polymeric and solvent stress contributions, $\tau_{P}+\tau_{S}$, and the pressure field, *p*,

(4) $$C_{D}=\frac{2}{\rho_{f}U^{2}A}\int_{\delta \Omega_{s}}\left(\tau_{P}+\tau_{S}-pI\right).n.x\;dS.$$

**Figure 2:**
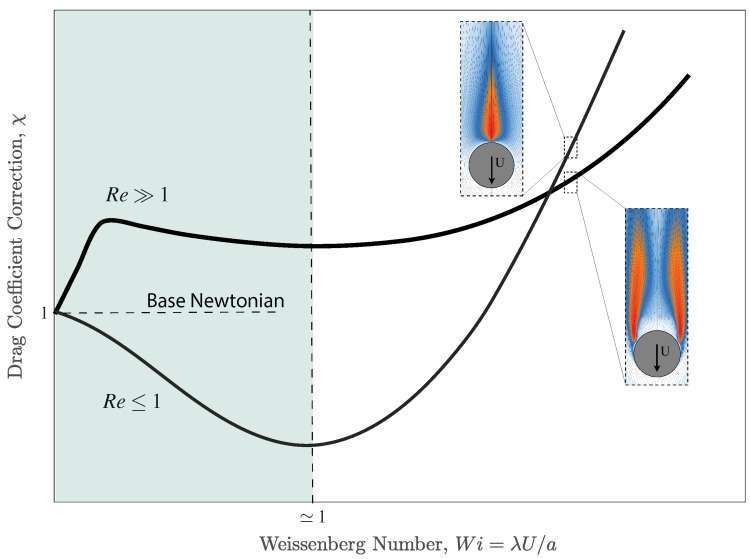
Schematic profiles of the drag correction coefficient vs. Weissenberg number for a particle translating through an unbounded viscoelastic fluid at different Reynolds numbers. The insets show the profile of the polymeric axial stress developed in the wake of the particle, i.e., the extent over which polymer chains are stretched due to the strong extensional flow (red and white colors show the maximum and minimum stresses, respectively).

In experimental studies, the drag coefficient can be calculated using the terminal velocity, *U*, measured for a sphere settling under the action of gravity, *g*, through a fluid. A relationship between the drag coefficient and terminal velocity can be deduced using the drag and gravitational force balance, leading to,

(5) $$\begin{array}{c} C_{D}=\frac{8ga}{3U^{2}}\left(\frac{\rho_{p}-\rho_{f}}{\rho_{f}}\right), \end{array}$$

where $\rho_{p}$ and $\rho_{f}$ are the density of the particle and fluid, respectively. Equations (4) and (5) at Re≪1 and zero elasticity, Wi=0, reduces to the base visco-inelastic (or Newtonian) value for the drag coefficient, namely CD=24Re [43]. At higher elasticity, the drag coefficient may increase or decrease depending on the flow conditions. Faroughi et al. [2] showed that at high Reynolds number, Re≫1, $C_{D}$ in a viscoelastic is always bigger than the base Newtonian value, as shown in Figure 2. At Re≤1, the drag coefficient of the particle first decreases (by a small amount) at low Weissenberg numbers, then bounces back at a critical Weissenberg number, and finally increases drastically due to large elastic stresses developing on the surface and in the wake of the particle. This phenomenon schematically shown in Figure 2 is also well reported in the literature [1,33]. The insets in Figure 2 show the profile of the polymeric axial stress developed in the wake of the particle at different Reynolds numbers. As depicted, at low Re, the polymer chains are stretched in the wake of particle with a maximum value close to the rear stagnation point where strong extensional flow is dominant. At high Re, due to the strong inertial effects, the axisymmetric flow past the particle shifts to a symmetry-breaking steady flow with a helical wake structure. The formation of symmetric eddies in the wake of particle relaxes the polymer chains on the center-line, and pushes the stress overshoot (maximum stretch) close to the flow separation points. The nonlinear inertial effects causing the formation of a steady axisymmetric toroidal eddy in the wake of the sphere greatly reduces the effect of elasticity on the drag coefficient. As illustrated in Figure 2, at high Weissenberg numbers, the monotonic enhancement of the drag coefficient arising from viscoelasticity is more pronounced for low Re flows. This is an important observation, as the elasticity effects at low Re regimes are more applicable to many industry operations (e.g., the proppant placement in the fracture networks [2,17]).

**Figure 3:**
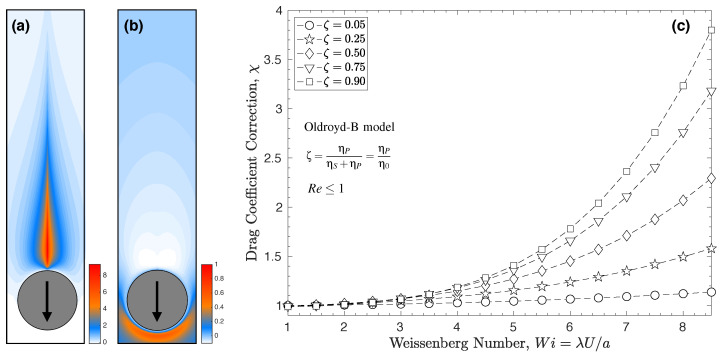
The effect of elasticity on the drag coefficient of a particle settling through a viscoelastic fluid. Panel (**a**,**b**) shows the contours of dimensionless polymeric stress components, $\tau_{P_{rr}}$ and $\tau_{P_{\phi \phi }}$ developed in the wake and front of the particle, respectively, at Re=0.1, Wi=2, and ζ=0.5 [20]. These stress components both increase with Wi and hinder the particle settling velocity, i.e., increase the drag coefficient of the particle. Contours are shown in the spherical polar coordinate frame, $\left\{r,\theta ,\phi \right\}$, on the r−θ plane where 0≤θ≤2π. The polymeric stress components are normalized by η0Ua. Panel (**c**) illustrates the relationship between the drag correction coefficient and Weissenberg number obtained from Equation (7) for an inertia-less spherical particle translating through an unbounded Oldroyd-B fluid with different polymeric retardation ratios.

Due to strong interactions of the fluid viscoelasticity and the complex kinematics of the mixed shearing and extensional flow around the particles, an exact solution to Equation (4) that performs well over a wide range of viscoelastic parameters is missing. Faroughi et al. [2] tackled this problem to a great extent using direct numerical simulation to parameterize the canonical behavior of the drag coefficient considering the strong interaction of viscoelasticity and kinematic parameters. They used Oldroyd-B model to parameterize the contribution of the polymer microstructural changes at a particle level to the momentum exchange between the mixture constituents of a dilute polymer solution. The Oldroyd-B model simply represents an elastic fluid with a constant viscosity, and hence elastic effects can be studied alone. This constitutive model is a good approximate model for Boger fluids made of a sufficiently viscous solvent, in which stresses due to the elasticity are quantifiable [34]. The Oldroyd-B model, however, may not be marginally accurate, especially in extreme extensional flow where the fictitious entropic spring allows for infinite stretching, i.e., infinite stress [1]. Faroughi et al. [2] observed a self-similarity of the evolution in the drag coefficient in the inertia-less flow regime, Re≤1, and fitted the numerical simulations in this regime to develop an explicit model for the drag coefficient correction. The model by Faroughi et al. [2] at Wi≤1 reduces to

(6) $$\begin{array}{c} \begin{array}{ccc} \chi =\frac{C_{D}}{\left(24/Re\right)}=1+\frac{1}{Wi^{4}+\left(6.288-6.111\zeta \right)Wi^{2}+0.0534}( & \left(0.06665\zeta -0.06392\zeta^{2}\right)Wi^{6} & +\\ & \left(-0.09422\zeta +0.07025\zeta^{2}\right)Wi^{4} & +\\ & \left(-0.00443\zeta +0.00248\zeta^{2}\right)Wi^{2} & ), \end{array} \end{array}$$

and at Wi≫1 reduces to

(7) $$\begin{array}{c} \begin{array}{ccc} \chi =\frac{C_{D}}{\left(24/Re\right)}=1+\frac{1}{Wi^{4}+\left(0.5014\zeta -0.02511\zeta^{2}\right)Wi^{2}}( & \left(0.0005713\zeta^{2}\right)Wi^{8} & +\\ & \left(0.0006+0.02517\zeta -0.02148\zeta^{2}\right)Wi^{6} & +\\ & \left(-0.02511\zeta +0.0009496\zeta^{2}\right)Wi^{4} & ), \end{array} \end{array}$$

for the drag coefficient correction, χ, which is predicted within 95% accuracy for Wi<5 and 0<ζ<1, see Figure 3. At low elasticity regime, Wi≤1, Equation (6) predicts very small reductions in drag for which χ≈1 is a safe assumption for practical applications. However, at high elasticity Wi>1, as shown in Figure 3c plotting Equation (7), the drag can be drastically enhanced and must be taken into consideration when comparing the carrying capacity of different fluids.

### 2.3. Rheological Properties

To determine the drag coefficient using Equations (6) and (7), one needs to glean the retardation ratio and relaxation time, among other rheological parameters, for dilute polymer solutions. The change in the zero-shear viscosity due to the presence of polymers of different types is relatively straightforward to measure using a conventional bulk rheometry [44,45,46] or using microfluidic viscometers [47,48]. Knowing the shear viscosity of the Newtonian solvent, $\eta_{S}$, one may characterize the Newtonian plateau region at lower shear rates, i.e., where the viscosity is independent of the shear rate, using a stress controlled shear rheometer, see for example Rubinstein and Colby [22], Kulicke and Clasen [49]. This method simply provides the zero-shear viscosity of the solution, $\eta_{0}$, using which the polymer contribution to the zero-shear viscosity, $\eta_{P}=\eta_{0}-\eta_{S}$, and the fluid’s retardation ratio, ζ, can be determined using Equation (2).

The determination of the relaxation time, λ, is not as simple as retardation ratio. Polymer solutions are usually best described using a spectrum of relaxation times accounting for relaxation processes occurring within the chain itself, as well as within the network of chains. For the shear flows of dilute polymer solutions, the determination of relaxation times poses several challenges. In these cases, the weak viscoelasticity signals can hardly be captured using conventional methods [50]. For an ideal dilute polymeric solution, the chain–chain interactions are absent, and the viscoelasticity of the fluid reduces to the viscoelasticity of isolated chains that still posses multiple relaxation processes related to the chain itself and the sub-chains on the backbone [22]. The relaxation process for the chain is slower than that of the sub-chains [50]. This suggests that the viscoelasticity of a dilute polymer solution, comprised of polymers with monodisperse molecular weight distribution, can be quantified by the longest relaxation time within the spectrum. The longest relaxation time is the time required for a isolated chain to relax from a stretched configuration to a random coil configuration [22,51]. Other modes with relaxation time smaller than the longest relaxation time do not appreciably contribute to the stress as they are not excited by the flow. The longest relaxation time strongly depends on both molecular weight and concentration of the polymer. Note that for low concentration and low molecular weight, measurements have to be performed at higher frequencies as the dominant dynamics gravitate to occur on shorter timescales [47]. Sometimes these frequencies are out of reach using conventional bulk rheometry due to the detection limit of the instrumentation caused by the onset of the inertial effects [47,52,53,54]. For example, the longest relaxation time is on the order of milliseconds and below for low-viscous water-based viscoelastic fluids [50,55]. In these scenarios, microfluidics has proven to be a promising tool to capture the correct modes of the dilute polymeric solutions [48].

## 3. Materials and Methods

### 3.1. Fluids and Preparation

Two different elastic fluids were used to investigate the influence of elasticity on the drag coefficient for slow flow around a sphere. The first fluid was composed of 0.1 wt.% polyacrylamide (5–6 MDa) dissolved in a solvent made of 90 wt.% glycerol and 10 wt.% DI water (this fluid is tagged as PAM/GLY for the rest of this paper). Polyacrylamide is known to adopt a relatively extended conformation in low salinity, and a random coil conformation in solutions containing high concentrations of ions. Deionized water is then used as a solvent to remove the reduction in extensibility of this polyacrylamide-based Boger fluid prepared for different measurements. The second fluid was composed of dissolving 16 wt.% high molecular weight polystyrene (20 MDa) in a solvent made of 70 wt.% low molecular weight polystyrene (500 Da) and 30 wt.% tricresyl phosphate (this fluid is tagged as PS/TCP for the rest of this paper). The mixture of tricresyl phosphate with low molecular weight polystyrene is known to be a good solvent for high molecular weight polystyrene, and results in a high extensibility for the solution [56,57]. For both fluids, conventional rheology experiments were conducted with several measurement geometries (cone-and-plate, parallel plate, concentric cylinder) to increase the range of shear rates. Each measurement is also repeated three times to ensure the integrity of the data. The rheology of the solutions was monitored as a function of time to be completely homogeneous (experimental error <2%) before measuring the final properties under the conditions of controlled room temperature at the same temperature as the falling sphere experiments (i.e., *T* = 20 ${}^{\circ }$C). For these solutions, $\eta_{0}$ and ζ are obtained using viscometric properties; the shear viscosity was measured as a function of shear rate fitted by the Carreau model [58,59]. The longest relaxation time was measured using the normal stress difference [60,61] and μ-rheometer [50] methods for PS/TCP and PAM/GLY, respectively.

### 3.2. Particle Settling Experiments

To probe a broad range of Weissenberg numbers, different Boger fluids possessing different relaxation times are needed. Due to the difficulty to formulate Boger fluids (i.e., polymer solutions with constant viscosity), various sizes and types of spherical particles were used with D=2a={0.5−12} mm and densities ρ={1300−7800}kgm3. All these particles are commercially available with a high precision, i.e., with diameter tolerance smaller than 25 μm, at Cospheric LLC, Santa Barbara, CA, USA. Using this set of spheres falling through the formulated solutions, it was possible to keep the nominal shear rates, accounting for wall decelerating effect, smaller than $\dot{\gamma }<50\;s^{-1}$, and explore the effect of Reynolds number and Weissenberg number on drag coefficient for a desired range of Re<1 and 0<Wi<8.5, respectively.

**Figure 4:**
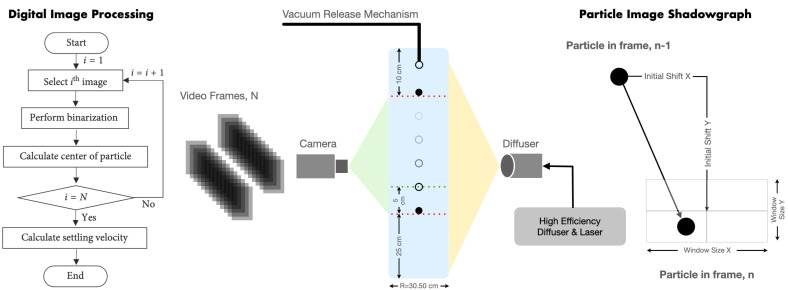
A schematic representation of the experimental setup and procedures to measure the settling velocity and drag coefficient of spherical particles translating through viscoelastic fluids.

The transient motion of a sphere along the center line of a glass cylindrical tube (with internal radius of *R* = 15.25 cm) is captured and measured using two techniques, (i) digital image processing (DIP) following Kim et al. [62], and (ii) particle image shadowgraph (PIS) following Arnipally et al. [23] to measure independent estimates of the steady-state terminal velocity of the particle settling through the Boger fluids. The DIP and PIS procedures to calculate the settling velocity are summarized in Figure 4. In DIP, binarization is used to differentiate target (particle) and background (viscoelastic fluid). After binarization, the particle region is converted to a set of white pixels, and the centroid of white pixels is marked to determine the position of the particle in each image. The displacement of the centroid, i.e., the difference of the position of the particle, in two consecutive images with a known time interval leads to the settling velocity. The PIS technique works based on the fundamental principal that shadow forms as light travels through different mediums of different refractive indices. The position of the particle in each image is determined depending on the shadow intensity. The settling velocity is then calculated using particle displacement in two consecutive images with known time interval as shown in Figure 4. Refer to Kim et al. [62] and Arnipally et al. [23] for measurement calibration and details about the required devices in these setups. The accuracy of both measurements are mostly limited to the spatial and temporal resolution of the image acquisition device and the light source to record the Lagrangian displacement of the falling sphere. It is important to note that releasing the sphere below the free surface right on the cylinder’s center line greatly affects the velocity data and particle trajectory. Therefore, the setup requires a reliable particle release mechanism on top of the cylinder. In this work, a firm vacuum mechanism was attached to a tube holding the particle at its end at around three particle diameter below the free surface. The reliability of measuring techniques and the accuracy of the data obtained are constantly checked against a Newtonian fluid. In our setup configuration, the confinement ratio is defined as the ratio of particle radius to cylinder radius varies in the range of a/R={0.0016−0.0384} for which the Fax́en correction factor [2],

(8) $$\begin{array}{ccc} f_{w}\left(\frac{a}{R}\right) & = & (1-2.10444\left(\frac{a}{R}\right)+2.08877{\left(\frac{a}{R}\right)}^{3}-0.94813{\left(\frac{a}{R}\right)}^{5}\\ & - & 1.372{\left(\frac{a}{R}\right)}^{6}+3.87{\left(\frac{a}{R}\right)}^{8}-4.19{\left(\frac{a}{R}\right)}^{10}+\ldots {)}^{-1}, \end{array}$$

approaches unity. Therefore, the effect of cylinder walls on the drag coefficient could be safely neglected. This assumption is in accordance with Arigo et al. [63] and provides us with the ability to directly compare the experimental results with Equation (7) developed for unbounded domain. Several calculations were also made using the theoretical analysis for the terminal velocity of the largest particles in the lowest viscosity fluid to obtain the right measurement window for the experiments assuring particle reaches its terminal velocity. Images were taken inside a window located 25 cm above the bottom of the cylindrical and 10 cm below the fluid free surface. A distance of 50 mm above the bottom line was found to satisfy all configurations reaching the steady-state velocity. Each velocity measurement is repeated five times, assuring the statistical accuracy and repeatability of the acquired data. In each velocity measurement, after cross-checking PIS and DIP methods, an average value of the velocities by these two methods was used in drag coefficient calculation. A relatively long time-interval (30 min) between measurements was considered to allow these elastic Boger fluids to fully relax to their stress-free state before running the next experiments [23].

### 3.3. Rheological Measurements

Both polymer solutions and their respective Newtonian solvents are characterized by a steady-shear flow procedure using a stress controlled shear rheometer (DHR-3 by TA Instrument using cone-and-plate, parallel plate, concentric cylinder geometries). All rheological measurements were performed at carefully controlled room temperature, *T* = 20 ${}^{\circ }$C.

**Figure 5:**
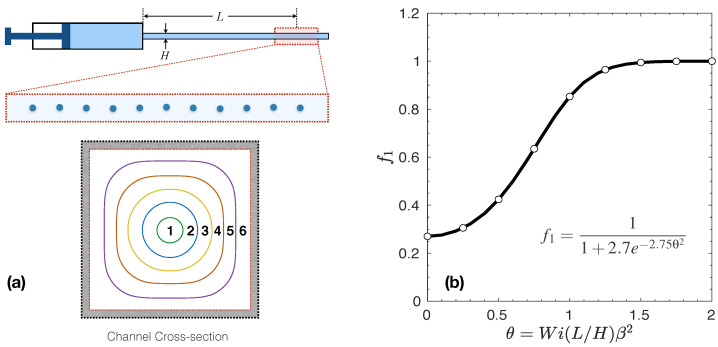
Schematic representation of a microfluidic-based device to measure the longest relaxation time of ultra-dilute and dilute polymer solutions. Panel (**a**) shows the μ-rheometer device that operates based on the transverse migration of solid particles in viscoelastic fluid flowing through a confined straight microchannel [54]. The bands marked on the cross-section of the channel is used to count particles trapped in those regions as they traveled a length of *L*, especially the fraction of particles aligned on the central band, $f_{1}$. Panel (**b**) shows the master curve for $f_{1}$ that can be directly used to approximate the longest relaxation time of ultra-dilute polymer solutions. This master curve does not need calibration for different geometrical setups and is universal for any viscoelastic fluids as long as Wi<0.5 within the rheometry experiments, and the confinement ratio between the suspended particle diameter, $D_{p}$, and channel cross-section diameter (or depth), *H*, is $D_{p}/H\leq 0.1$.

The longest relaxation time for the PAM/GLYC system could not be measured using conventional rheometry due to technical limitations of the instruments. Instead, the μ-rheometer method, i.e., a microfluidic rheometer, was used for the measurements of the longest relaxation time [50,54]. The working principle of the μ-rheometer is based on the transverse migration of solid particles occurring when the suspending viscoelastic fluid flows under an inertia-less Poiseuille flow through a confined straight microchannel. In this method, the fraction of particles aligned on the center-line, i.e., $f_{1}$ moving through the band number 1 as shown in Figure 5a, which is measured experimentally using optical microscopy at a distance, namely *L*, from the inlet position. This step is performed by dividing the cross-section of the microchannel arbitrarily into six bands for which the average velocities and cross-sectional area are calculated, see Del Giudice et al. [54] for more details. Knowing the expected velocities for particles in each band calculated from the fluid velocity, one can apply a particle tracking method to calculate the normalized fraction of particles in the first band using,

(9) f1=N1A1V1ΣNkAkVk,

where $N_{k}$ represents the number of particles flowing in the band *k*, and $V_{k}$ and $A_{k}$, respectively, show the average velocity and the cross-sectional area of the fluid enclosed in the kth band. For a given set of geometrical parameters (e.g., the channel cross-section diameter, *H*, and the confinement ratio defined as $\beta =D_{p}/H$ where $D_{p}$ is the diameter of the suspended particles), once $f_{1}$ is measured, the θ value can be calculated using the master curve shown in Figure 5b. The θ value can be then translated to the longest relaxation time of the solution knowing $\theta =Wi\left(L/H\right)\beta^{2}$ [54].

It is important to note that the master curve plotted in Figure 5a does not need calibration for different geometrical setups and is universal for any viscoelastic fluids as long as the Weissenberg number of the flow is kept below Wi<0.5 (within the rheometry experiments), and the confinement ratio is equal or smaller than 0.1, together corresponding to θ≤1.4. For a cylindrical microchannel with diameter *H*, the following equation,

(10) $$\begin{array}{c} \lambda =\frac{\pi }{4}\frac{1}{\beta^{2}}\frac{H^{4}}{LQ}\sqrt{\frac{1}{2.75}ln\left(\frac{2.7f_{1}}{1-f_{1}}\right)}\;, \end{array}$$

can be deduced for the longest relaxation time of dilute polymer solutions using the aforementioned procedures to evaluate $f_{1}$. Here, the characteristic shear rate in Wi number is replaced by $\dot{\gamma }=4Q/\pi H^{3}$ in which *Q* represents the imposed volumetric flow rate. Here, specifically, polystyrene particles having a 10 μm diameter (Polysciences Inc) were added to the PAM/GLY solutions at a mass concentration ϕ=0.01 wt%. Flowing particles were observed using an inverted microscope (Zeiss Axiovert), while videos were recorded with a high-speed camera (Photron Mini UX50). The flow rate was controlled using a pressure pump (Dolomite Microfluidics). The resulting videos were analysed using a particle tracking software subroutine in IDL [64].

## 4. Results and Discussion

### 4.1. Fluids Rheology

**Figure 6:**
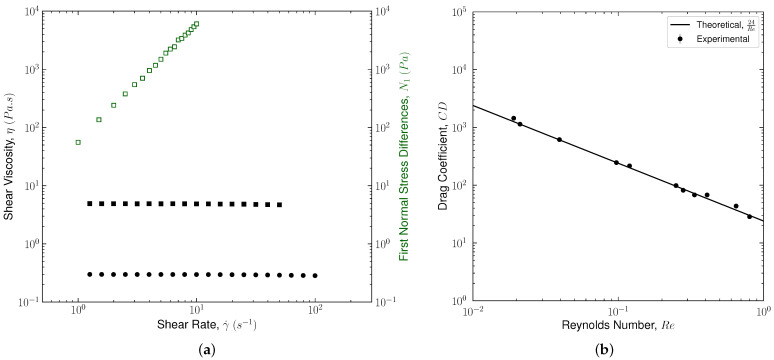
Panel (**a**) shows the results of the shear viscosity (filled circles for PAM/GLY fluid and filled squares for PS/TCP fluid) and the first normal stress difference $N_{1}$ (empty squares for PS/TCP fluid) as a function of shear rate. Panel (**b**) shows the comparison between theory and experimental results obtained for the drag coefficient of a spherical particle translating through a Newtonian fluid at Re≤1. The solvent for the PS/TCP fluid is used as the test Newtonian fluid possessing $\eta_{s}$ = 2.17 (Pa.s). The errorbars account for the dispersion around the average value of the drag coefficient measured experimentally. The mean value of the relative standard deviation for all measurements, conducted at *T* = 20 ${}^{\circ }$C and repeated five times, was less than 0.10%.

Results of the bulk shear rheology measurements are shown in Figure 6a. For the PS/TCP solution, the fluid was designed to stay within the semi-dilute regime, $c>c^{*}$. Here, $c^{*}$ is the overlap concentration defined as [22],

(11) $$\begin{array}{c} c^{*}=\frac{M_{w}}{\frac{4}{3}\pi N_{A}R_{g}^{3}}≃\frac{0.77}{\left[\eta \right]}, \end{array}$$

where $N_{A}$ is Avogadro’s constant, $R_{g}$ is the radius of gyration, and [η] denotes the intrinsic viscosity that depends on the molar mass of the chain, the degree of polymer chain branching, as well as the type of solvent in which the polymer is dissolved [22]. The overlap concentration is used to distinguish the onset of semi-dilute and eventually entangled regimes. At $c>c^{*}$, the polymer solution viscoelasticity is not only related to the individual chain contributions, but to the developed network of coils that dramatically changes the solution behavior under different flow conditions [49,65]. The shear viscosity and first normal stress difference, $N_{1}$, are reported in Figure 6a for the PS/TCP solution. For the PAM/GLY solution, the fluid was designed to stay within the dilute regime, $c<c^{*}$. In this regime, the individual polymer chains are far placed and rare hydrodynamic, steric, or frictional interactions present [24]. Therefore, the viscoelasticity of the solution is mostly associated with the viscoelasticity of individual polymer coils summed linearly. For the PAM/GLY solution, only the shear viscosity as a function of the shear rate is reported. Due to the detection limit of the rheometer in this regime, a reliable measurement for $N_{1}$ was not produced.

Both polymer solutions maintain a constant viscosity, as one of the most important characteristics of the Boger fluid, within the full range of shear rates present in the settling experiments at $\dot{\gamma }<50\;\left(1/s\right)$. The zero-shear viscosity, $\eta_{0}$, and the polymer contribution to the solution viscosity, $\eta_{p}$, are determined by fitting the Carreau model [40],

(12) $$\begin{array}{c} \eta =\eta_{\infty }+\left(\eta_{0}-\eta_{\infty }\right){\left(1+{\left(\lambda \dot{\gamma }\right)}^{2}\right)}^{\frac{n-1}{2}}, \end{array}$$

to the viscosity data shown in Figure 6a. In Equation (12), $\eta_{\infty }$ is the plateau viscosity at infinite shear rate, and *n* is the flow index accounting for the shear-thinning of the solution. For Boger fluids, the flow index should approach unity, n→1. The λ, here, has a unit of time and generally corresponds to the inverse of the shear rate at which the turnover occurs between the Newtonian plateau and the shear-thinning region [66]. From the best fit, at $c>c^{*}$, λ in Equation (12) can be used as an estimate of the longest relaxation time. This conclusion is based on the fact that the polymer chains are not in a random coiled configuration anymore and start to stretch at the onset of shear-thinning features. The best fit of Equation (12) to the PS/TCP viscosity data shown in Figure 6a leads to $\eta_{0}$ = 4.32 (Pa.s) and $\eta_{p}$ = 2.15 (Pa.s) with $R^{2}=0.987$. The best fit to PAM/GLY viscosity data shown in Figure 6a leads to $\eta_{0}$ = 0.31 (Pa.s) and $\eta_{p}$ = 0.22 (Pa.s) with $R^{2}=0.992$.

For the PS/TCP solution, the longest relaxation time is determined using the first normal stress difference. $N_{1}$. This is a well-practiced approach to determine an approximate value for the longest relaxation time of polymer solutions at $c>c^{*}$ [67]. This is because, under shear flow, the polymers dissolved in the base fluid tend to align with the flow streamlines, while they inherently tend to come back to their undisturbed conformation. These chain-level interactions lead to an extra tension in the direction of the flow attributed to the fluid elasticity. Normal stresses are zero for Newtonian fluids, i.e., $N_{1}=0$. Normal stresses thus could be used as a measure to obtain the level of elasticity, and hence, the relaxation time for polymeric (viscoelastic) fluids. All nonlinear constitutive models of viscoelastic fluids provide an expression to predict normal stress differences [60]. For a Boger fluid represented by Oldroyd-B model under a steady shear flow, $N_{1}$ can be determined as,

(13) $$\begin{array}{c} N_{1}=2\eta_{0}\lambda \zeta {\dot{\gamma }}^{2}, \end{array}$$

which is linear in both λ and ζ [60,68]. At a known value of $\zeta =\eta_{P}/\eta_{0}$, fitting Equation (13) to the measured $N_{1}$ data (see Figure 6) leads to the longest relaxation time of the polymer solution. The best fit of Equation (13) to the $N_{1}$ data reported for PS/TCP solution leads to λ=2.463 (s) with $R^{2}=0.996$.

For the dilute PAM/GLY solution where $c<c^{*}$, the characterization of elasticity effects and relaxation times is beyond the range measurable in the conventional geometries used in most of the shear and extensional rheometers [50]. Therefore, the $N_{1}$ method hardly provides reliable estimation for λ as it is difficult to ensure the integrity of the experimental data [68,69,70]. In these cases, a very rough method to provide an approximate value for the longest relaxation time is to use the Zimm theory [22]. This theory assumes the longest relaxation time is independent of the polymer concentration in a very dilute polymer solutions. However, this assumption is not valid under all flow conditions. For example, under strong extensional flow such as flow past a particle, polymer coils become substantially stretched resulting in an increased volume of interaction, which causes the overlap to happen at polymer concentration much below the $c^{*}$. Clasen et al. [24] concluded that the longest relaxation time depends on the polymer concentration even at cc*<1, but this dependency truly vanishes at cc*<0.01, known as ultra-dilute polymer solutions, regardless of how much polymer chains are deformed beyond their equilibrium state. For 0.01≤cc*≤1, the longest relaxation time is shown to exhibit a power-law scaling with the reduced concentration, cc*, where the magnitude of the exponent depends on the thermodynamic quality of the solvent [24]. Several methods have been proposed to glean the longest relaxation time for this region [55], from which those based on microfluidics are shown to outperform the others to estimate the relaxation time of viscoelastic fluids, down to milliseconds [53,54]. As described in Section 3.3, the newest microfluidics method is the μ-rheometer [50] This method is utilized to obtain the longest relaxation time for the dilute PAM/GLY solution in this work. The μ-rheometer approach predicted λ=0.023 s for this solution, which is smaller by two orders of magnitude than the value predicted for the PS/TCP solution.

These PAM/GLY and PS/TCP fluid choices provide the possibility to experimentally explore the effect of viscoelasticity on the drag coefficient of a sphere settling in both weakly elastic fluid flows, Wi≤1, and highly elastic fluid flows, Wi>1. Table 1 summarizes the rheological results for both PAM/GLY and PS/TCP solutions.

### 4.2. Drag Measurements

Conducting the particle settling experiments using viscoelastic fluids is challenging. For example, at a low Weissenberg number, the changes in the drag coefficient may well be within the experimental error. At a high Weissenberg number, the polymer chains may not relax to their stress-free condition if experiments are not well-spaced temporally. Therefore, to generate statistically significant data, a highly calibrated setup for velocity measurement (as shown in Figure 4) is needed in addition to a multitude of measurement repetitions. In this work, all the settling experiments are conducted at a constant room temperature, *T* = 20 ${}^{\circ }$C, unless otherwise stated. The experimental setup and velocity measurement procedures (DIP and PIS) are constantly calibrated by comparing the drag coefficient of spheres with different densities in an asymptotically unbounded Newtonian fluid at Re≤1. For this purpose, spherical particles with different characteristics (i.e., types, diameters, and densities) were used as summarized in Table 2. The solvent for the PS/TCP fluid (i.e., 70 wt.% low molecular weight polystyrene and 30 wt.% tricresyl phosphate) was also used as the test Newtonian fluid possessing $\eta_{s}=2.17$. The terminal velocity, *U*, measured for a sphere settling under the action of gravity in this Newtonian fluid was measured using DIP and PIS methods, converted to the drag coefficient using Equation (5) and finally compared with $C_{D}=24/Re$. For each particle, the velocity (and hence the drag coefficient) measurement is repeated five times. A sample result for this calibration process is shown in Figure 6b, where a good agreement between the experimental data and the universal drag coefficient is observed at a different Reynolds number (Re<1). The errorbars in Figure 6b account for the dispersion around the average value of the drag coefficient measured experimentally. The mean value of the relative standard deviation for all measurements, conducted at *T* = 20 ${}^{\circ }$C and repeated five times, was less than 0.10%. This small deviation is greatly attributed to the small temperature tolerance, and possibly the particle release mechanism.

In viscoelastic fluids, as discussed in Section 2, the drag coefficient not only changes with Re, but also varies as a function of the Weissenberg number and retardation ratio, i.e., CD=f(Re,Wi,ζ). The effect of Wi and ζ on the drag coefficient of a spherical particle at Re<1 was experimentally studied using the calibrated experimental setup and measurement procedures. Here, to quantify the effect of Weissenberg number, spherical particles with different characteristics were used to achieve 0<Wi<8.5. These particles were carefully selected to produce (i) a shear rate below than 50 (1/s) to stay in the constant-viscosity flow regime, and (ii) a Reynolds number below than unity (Re<1) for both PAM/GLY and PS/TCP solutions. The characteristics of the particles, and the associated ranges of Wi, Re, and $\dot{\gamma }$ obtained for each family of particles, are reported in Table 2.

**Figure 7:**
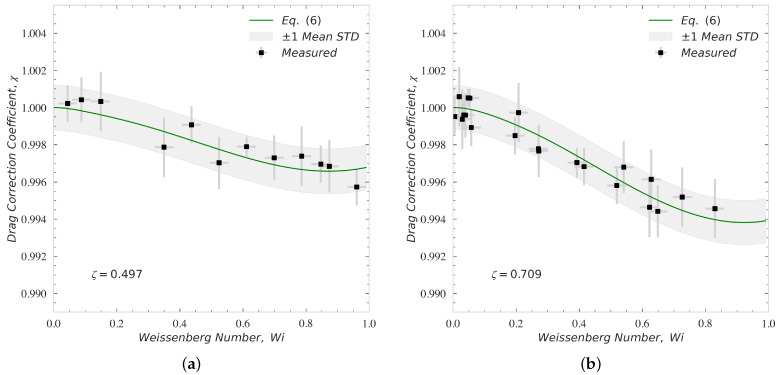
The comparison between measured and theoretical drag correction coefficient for particles settling through an asymptotically unbounded viscoelastic fluid at Re<1 and Wi<1. Panel (**a**) shows the comparison for PS/TCP solution, and Panel (**b**) shows the comparison for PAM/GLY solution. The errorbars account for the dispersion around the average value of the drag coefficient and Wi measured experimentally (5 measurements were carried out for each Wi value to assure the repeatability). The shaded area highlights a region constructed using the theoretical values of χ (Equation (6)) ±σ, where σ denotes the mean standard deviation for all measurements conducted for each solution.

Figure 7 shows the comparison between the measured and theoretical drag correction coefficient, χ, for particles settling through an asymptotically unbounded fluid at low Reynolds numbers, Re<1, and low Weissenberg number, Wi<1. The theoretical drag correction coefficients are calculated from Equation (6). This comparison is shown for the PS/TCP solution with ζ=0.497 in Figure 7a, and for the PAM/GLY solution with ζ=0.709 in Figure 7b. As expected from the literature [1,2,33] and Equation (6), using a carefully measured relaxation times and other rheological properties, the calibrated velocity measurement procedures captured a slight reduction in drag coefficient for both values of ζ at Wi<1 and Re<1. In these measurements, again, each settling test is repeated five times assuring the statistical accuracy and repeatability of the acquired data. The vertical errorbars account for the dispersion around the average value of the drag coefficient correction measured experimentally at each Wi. The horizontal errorbars account for the dispersion around the average value of Weissenberg number attributed to the tolerance of sphere diameters and shear rate measurement. The shaded area highlights a region constructed using the theoretical values of χ (Equation (6)) extended by ±1σ, where σ denotes the mean standard deviation for all measurements conducted at Wi<1. The mean value of the relative standard deviation is 0.11% for the PS/TCP solution and 0.12% for the PAM/GLY solution. Figure 7 shows that increasing the polymer viscosity, i.e., the retardation ratio, results in a more pronounced reduction of the drag coefficient at Wi<1.

**Figure 8:**
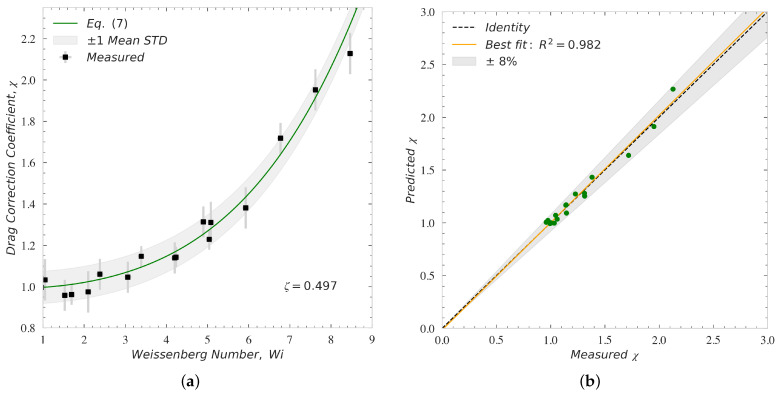
Panel (**a**) shows the comparison between measured and calculated drag correction coefficient for particles settling through an asymptotically unbounded PS/TCP solution at Re<1 and Wi>1. The errorbars account for the dispersion around the average value of the drag coefficient and Wi measured experimentally (5 measurements were carried out for each Wi value to assure the repeatability). The shaded area highlights the region where the theoretical values are extended by ±σ. Here, σ denotes the mean standard deviation for the measurements. Panel (**b**) shows the comparison between measured drag coefficient correction and predicted drag coefficient corrections of particles calculated using Equations (6) and (7). The shaded area in panel (**b**) highlights the bounds encompassing all the data by extending the identity line, x=y, by ±8%.

At higher Weissenberg numbers, no data was produced for the PAM/GLY solution due to its very low relaxation time and the limitation on the particle size satisfying $\dot{\gamma }<50$ (1/s) and Re<1. However, for the PS/TCP solution that possess a high zero-shear viscosity and a large relaxation time, a broad range for Wi was achieved using the particles listed in Table 2 while satisfying all other kinematic constraints. Figure 8a shows the comparison between the measured and theoretical drag correction coefficient, χ, for particles settling through an asymptotically unbounded PS/TCP solution at Re<1 and Wi>1. As expected from the literature [2,23] and Equation (7), the drag coefficient for a spherical particle increases, i.e., χ>1. The experimental results follow Equation (7) very well at Re<1. The vertical and horizontal errorbars account for the dispersion around the average value of the measured drag coefficient correction and Wi, respectively. The horizontal errorbars are smaller than the symbols and thus are masked in Figure 8a. The shaded area highlights a region constructed using the theoretical values of χ (Equation (7)) extended by ±1σ, where σ denotes the mean standard deviation for all measurements conducted. The mean value of the relative standard deviation is 5.97% for all measurements conducted in the PS/TCP solution at Wi>1. The mean value of the relative standard deviation in this case is one order of magnitude larger than its counterpart obtained for Newtonian and slightly elastic fluids (Wi<1). The increase in the relative standard deviation measured at high Weissenberg numbers might be attributed to (i) higher elasticity effects pushing the particle off the flow centerline where it starts to rotate, (ii) repetitive experiments causing large and continual disturbance of the polymer chains located on the flow centerline. Figure 8b illustrates the comparison between measured drag coefficient correction and predicted drag coefficient corrections calculated using Equations (6) and (7). The best fit to the data yields $R^{2}=0.982$. The shaded area highlights the bounds encompassing all the data by extending the identity line, x=y, by ±8%.

The experimental data plotted in Figure 7 and Figure 8 are unique and were not reported previously in the literature. These data were captured using carefully designed elastic fluids and velocity measurement procedures. These datasets show the presence of an initial reduction (i.e., second order decrease in Wi≤1), as well as a large enhancement (i.e., higher order increase in Wi>1) in the viscoelastic drag coefficient due to elasticity. These data also confirm that the approximate model, Equations (6) and (7), developed based on direct numerical simulation of Oldroyd-B fluids past a sphere, can confidently predict the effect of elasticity on the viscoelastic drag coefficient at 0<Wi<8.5. This moderate range of Weissenberg number is typically experienced by particles in dilute polymeric fracturing fluids, with λ≈O(100 ms), flowing through the fracture networks with $\dot{\gamma }\approx O\left(100\;1/s\right)$; see, for example, Malhotra and Sharma [71] and Hu et al. [72]. Therefore, this numerically-driven model can be used to rapidly compare the particle-carrying capacity of different polymeric fluids.

When polymeric solutions are strongly shear-thinning, the effect of elasticity on the drag coefficient reduction or enhancement can be masked [12,23]. Shear-thinning behavior leads to more complex and nonlinear dependencies at non-vanishing Weissenberg numbers. Very recently, Faroughi et al. [13] numerically studied the coupled effects of elasticity, shear-thinning, and inertia on the viscoelastic drag coefficient correction. When considering a strong shear-thinning behavior, they showed that increasing inertia (i.e., Re number) and elasticity (increasing the Wi number) of the flow lead to a strong reduction in the viscoelastic drag coefficient correction. These effects not only mask the enhancement due to elasticity (e.g., the one observed in Figure 8 for a Boger fluid), but also decrease it sharply to a value lower than unity (χ<1). The shear-thinning effects on the particle transport, however, have not been comprehensively quantified, and require further investigations.

## 5. Conclusions

In this work, we performed an experimental campaign using different polymer solutions and rheological techniques to validate a theoretical model introduced previously [2] to describe particle settling in viscoelastic liquids with negligible shear-thinning. With this aim, we employed two Boger fluid formulations with distinct longest relaxation time values, and spherical particles with different characteristics were used to quantify the effect of Weissenberg number (i.e., elasticity) on the drag coefficient in 0<Wi<8.5 at Re<1. The drag coefficient decreases with Wi at a low level of elasticity (Wi<1), and increases with Wi at a high level of elasticity (Wi>1). The comparison between the measured and calculated drag coefficient data collectively yields $R^{2}=0.982$, endorsing the accuracy of the approximate model for the range studied here. Our experimental results also show a self-similarity in the evolution of the drag coefficient with elasticity in the inertia-less flow regime. Future work should focus on the combined effect of fluid shear-thinning, elasticity, and inertia on the particle settling behavior, a problem that is not still fully understood and quantified.

## Figures and Tables

**Table 1:** Fluid rheological characteristics. For the PS/TCP solution, the longest relaxation time is determined by fitting the Oldroyd-B model expressed by Equation (13) to the data measured for $N_{1}$ shown in Figure 6a. For the PAM/GLY solution, the longest relaxation time is determined using the μ-rheometer approach. The zero-shear viscosity, $\eta_{0}$, and the solvent and polymer contributions to the viscosity, $\eta_{S}$ and $\eta_{P}$, are determined by fitting Equation (12) to the viscosity data shown in Figure 6a.

Boger Fluids	$\rho_{f}$ (kg/m${}^{3}$)	$\eta_{0}$ (Pa.s)	$\eta_{S}$ (Pa.s)	$\eta_{P}$ (Pa.s)	ζ (-)	λ (s)
PAM/GLY	1242.1	0.31	0.09	0.22	0.709	0.023
PS/TCP	1162.8	4.32	2.17	2.15	0.497	2.463

**Table 2:** Spherical particle characteristics used in settling measurements.

Material	Density (kg/m${}^{3}$)	Diameter (mm)	$\dot{\gamma }$ (1/s)	Re	Wi
Cellulose Acetate	1300	1.0–12.0	<4.0	<1.0	<1.0
White Polymer	1800	5.0–6.0	<13	<1.0	<2.5
Soda Lime Glass	2500	1.0–8.0	<45	<1.1	<8.5
Yttria Zirconia	6000	0.5–1.6	<29	<0.2	<5.2
Stainless Steel	7800	0.5–1.2	<30	<0.1	<5.5

## Data Availability

Data can be found at https://gilab.wp.txstate.edu/research/ (accessed on 4 February 2022).
